# Supplementary material for: Early Immunological Effects of Ischemia-Reperfusion Injury: No Modulation by Ischemic Preconditioning in a Randomised Crossover Trial in Healthy Humans
Source: Int J Mol Sci. 2019 Jun 13;20(12):2877. doi: 10.3390/ijms20122877 (PMC6628232; doi:10.3390/ijms20122877)
Supplement: Supplementary file 1 [file ijms-20-02877-s001.pdf]

International Journal of
Molecular Sciences

MDPI

Supplementary

Gating strategy for immune cell subsets and the intracellular markers

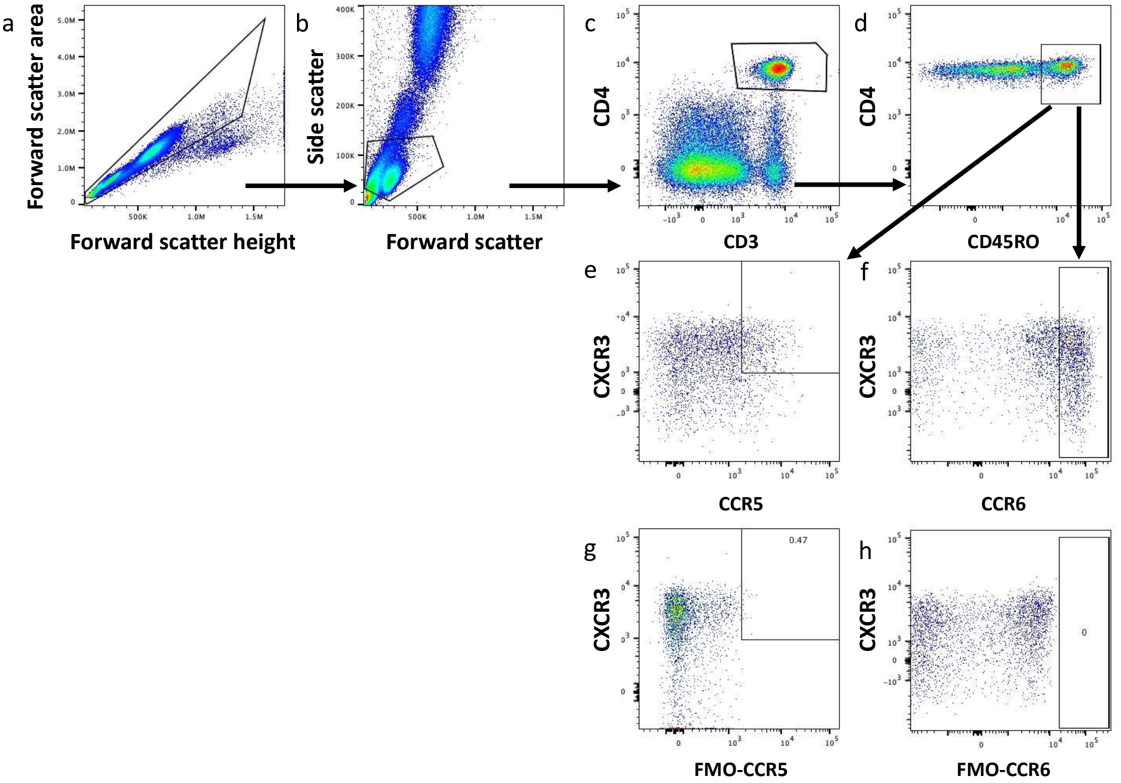

Figure 1. Gating of Th1 and Th17 cells. A: Cells were gated in an FSC-H and FSC-A dot plot to eliminate doublets. B: Lymphocytes were gated in an FSC vs. SSC dot plot. C: CD3+/CD4+ cells were gated. D: CD45RO+ cells were chosen to analyse memory Th-cells. E: CCR5+/CXCR3+ cells were gated on the basis of a CCR5-FMO control shown in G. CCR5+/CXCR3+ were used as markers for Th1 cells. F: CCR6+ cells were gated on the basis of a CCR6-FMO control shown in H. CCR6+ were used as marker for Th17 cells. G: FMO control of CCR5 showing 0,47% of cells in the FMO-CCR5 gate. H: FMO control of CCR6 showing 0% cells in the FMO-CCR6 gate. FSC: Forward scatter. FSC-H: Forward scatter height. FSC-A: Forward scatter area. SSC: Side scatter. FMO: Fluorescence-minus-one control.

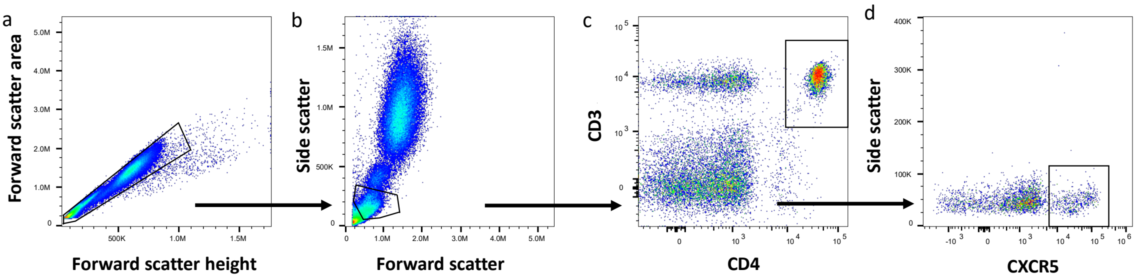

Figure 2. Gating of Tfh cells. A: Cells were gated in an FSC-H and FSC-A dot plot to eliminate doublets. B: Lymphocytes were gated on an FSC vs. SSC plot. C: CD3+/CD4+ cells were chosen. D:

CXCR5+ cells were used as markers for Tfh-cells. FSC: Forward scatter. FSC-H: Forward scatter height. FSC-A: Forward scatter area. SSC: Side scatter.

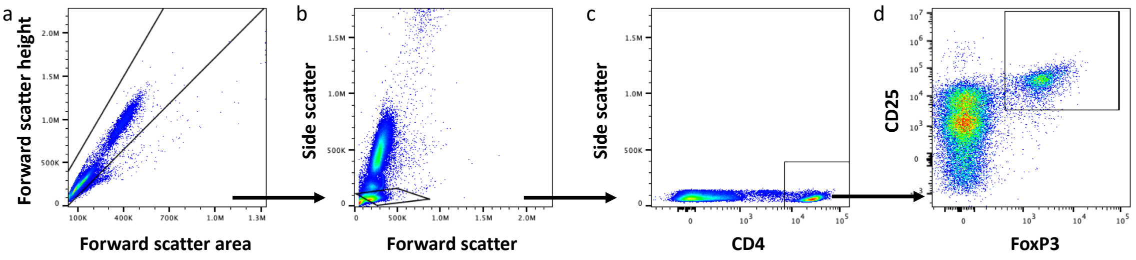

Figure 3. Gating of Tregs (CD25+/FoxP3+). A: Cells were gated in an FSC-H and FSC-A dot plot to eliminate doublets. B: Lymphocytes were gated on an FSC vs. SSC plot. C: CD4+ cells were chosen. D: CD25+ and FoxP3+ cells were used as markers for Tregs. FSC: Forward scatter. FSC-H: Forward scatter height. FSC-A: Forward scatter area. SSC: Side scatter.

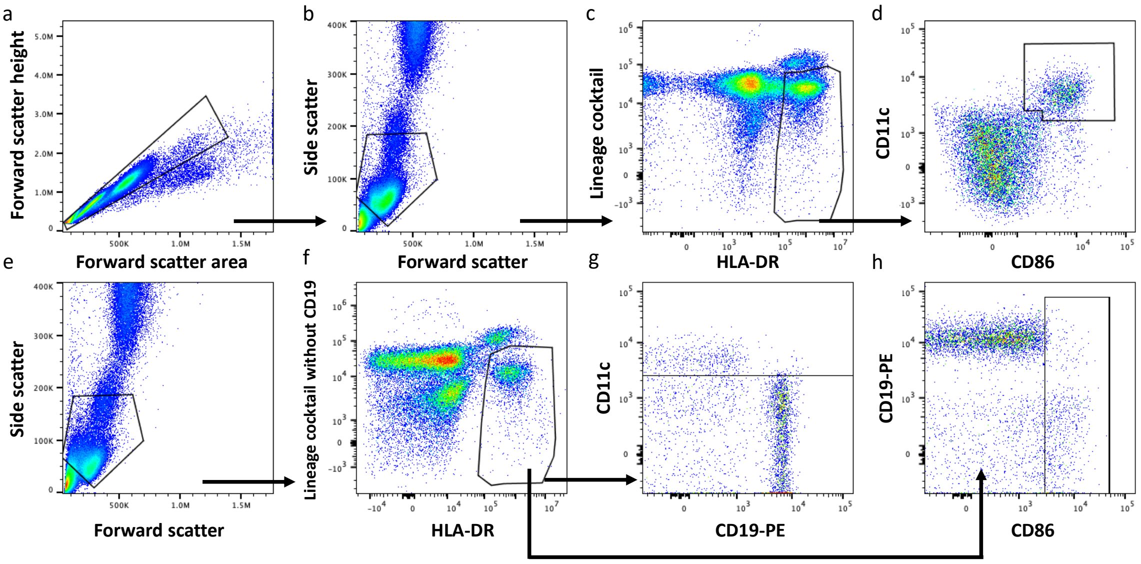

Figure 4. Gating of mDCs. mDC-gating strategy. A: Cells were gated on an FSC-H and FSC-A plot to eliminate doublets. B: DCs were gated on an FSC vs. SSC dot plot. C: Negative lineage cocktail and positive HLA-DR were chosen. D: CD86+ and CD11c+ were used as markers for mDC. E-H: ILT3-PE were substituted with CD19-PE to clarify, that B-cells were not included in our gating of mDCs. E: Cells were gated on an FSC-H and FSC-A plot to eliminate doublets. F: DCs were gated on an FSC vs. SSC dot plot. G and F: Gating was performed to tailor our gating for mDCs in D. G: Elucidated that our gating of positive CD11c cells did not include CD19+ cells. H: Clarified that our gating of positive CD86 cells included a minimum of CD19 cells (< 2%). Tailoring the gating for mDCs on the basis of G and H it was fair to say, that no significant amount of CD19+ cells were to be found in our mDC gating in D. FSC: Forward scatter. FSC-H: Forward scatter height. FSC-A: Forward scatter area. SSC: Side scatter. DCs: Dendritic cells. mDCs: Myeloid dendritic cells.

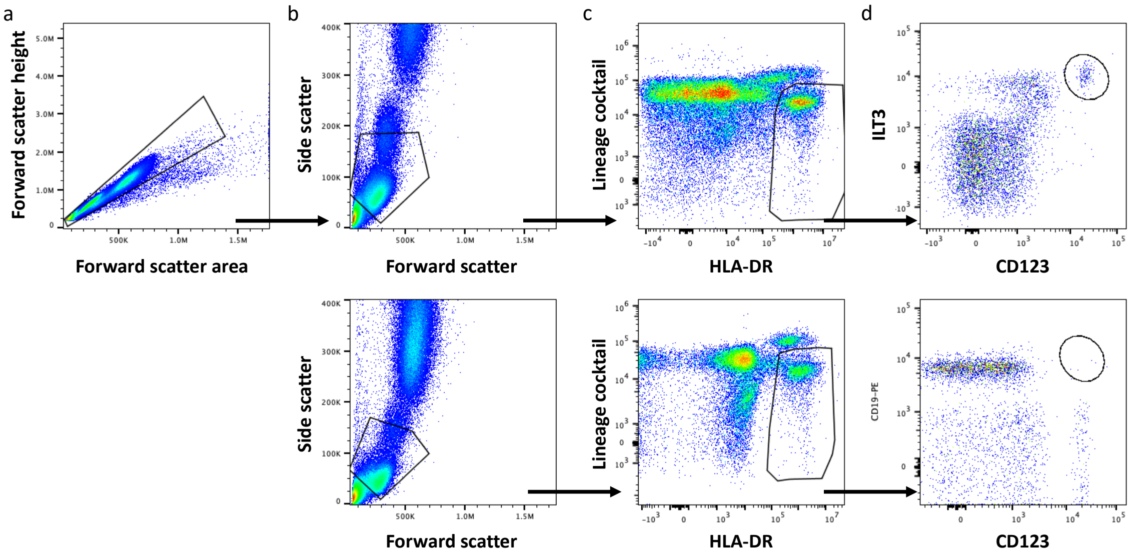

Figure 5. Gating of pDCs. Gating strategy for pDCs. A: Cells were gated on an FSC-H and FSC-A plot to eliminate doublets. B: DCs were gated on an FSC vs. SSC dot plot. C: Negative lineage cocktail and positive HLA-DR were chosen. D: CD123+ and ILT3+ were used as markers for pDC. E-G: ILT3-PE were substituted with CD19-PE to clarify, that B-cells were not included in our gating of pDCs. E: Cells were gated on an FSC-H and FSC-A plot to eliminate doublets. F: DCs were gated on an FSC vs. SSC dot plot. G: Gating was performed to secure our gating for pDCs in D, as we showed that gating positive for CD123 did not have a relevant amount of CD19+ cells (< 0,2%). Based on G it was fair to say, that no significant amount of CD19+ cells were found in our pDC gating in D. FSC: Forward scatter. FSC-H: Forward scatter height. FSC-A: Forward scatter area. SSC: Side scatter. DCs: Dendritic cells. pDCs: Plasmacytoid dendritic cells.

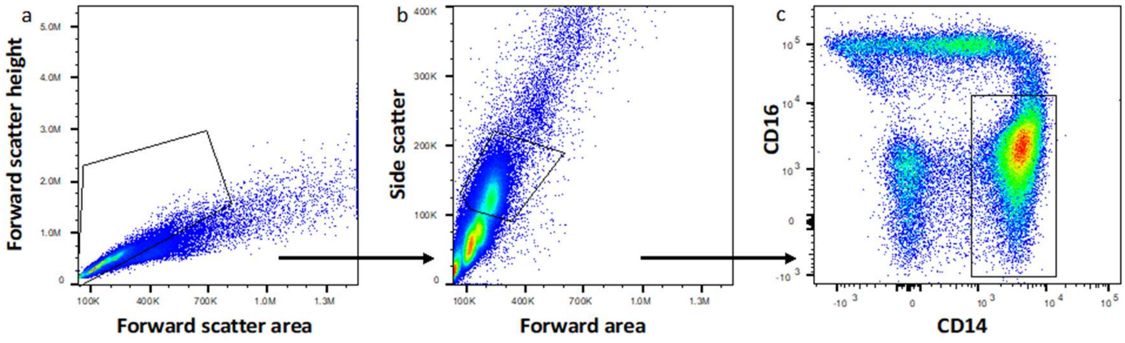

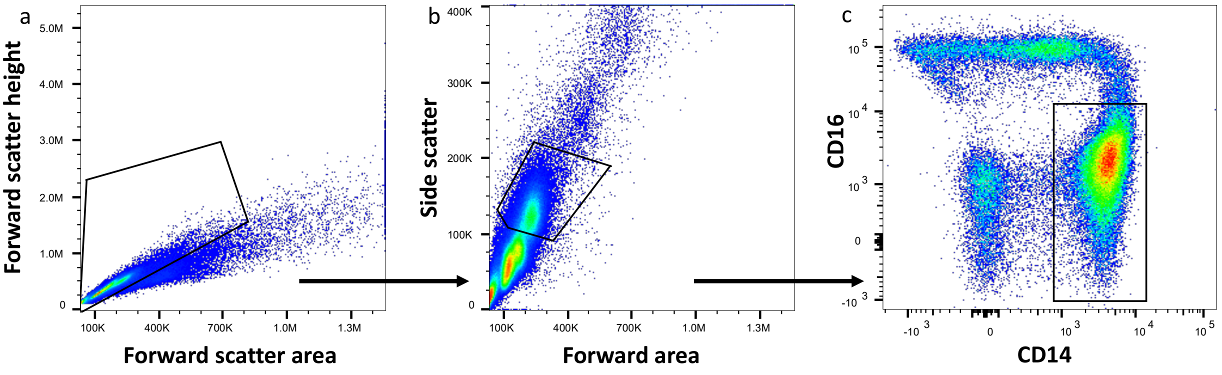

Figure 6. Gating of Classical monocytes (CD14++/CD16-). A: Cells were gated in an FSC-H and FSC-A dot plot to eliminate doublets. B: Monocytes were gated on an FSC vs. SSC plot. C: CD14++/CD16- were used as markers for classical monocytes. FSC: Forward scatter. FSC-H: Forward scatter height. FSC-A: Forward scatter area. SSC: Side scatter.

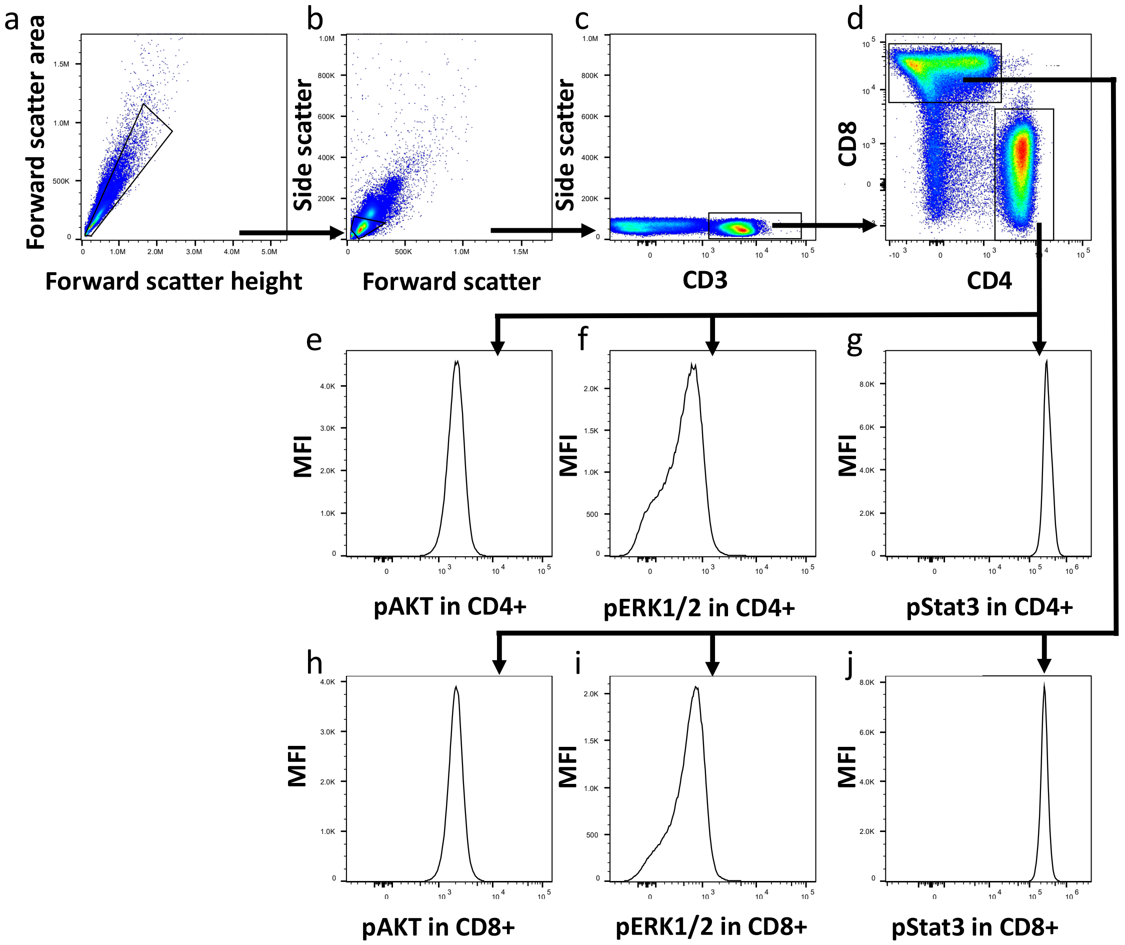

a
b
c
d
Forward scatter area
Forward scatter height
Side scatter
Forward scatter
Side scatter
CD3
CD8
CD4
e
f
g
MFI
pAKT in CD4+
pERK1/2 in CD4+
pStat3 in CD4+
h
i
j
pAKT in CD8+
pERK1/2 in CD8+
pStat3 in CD8+

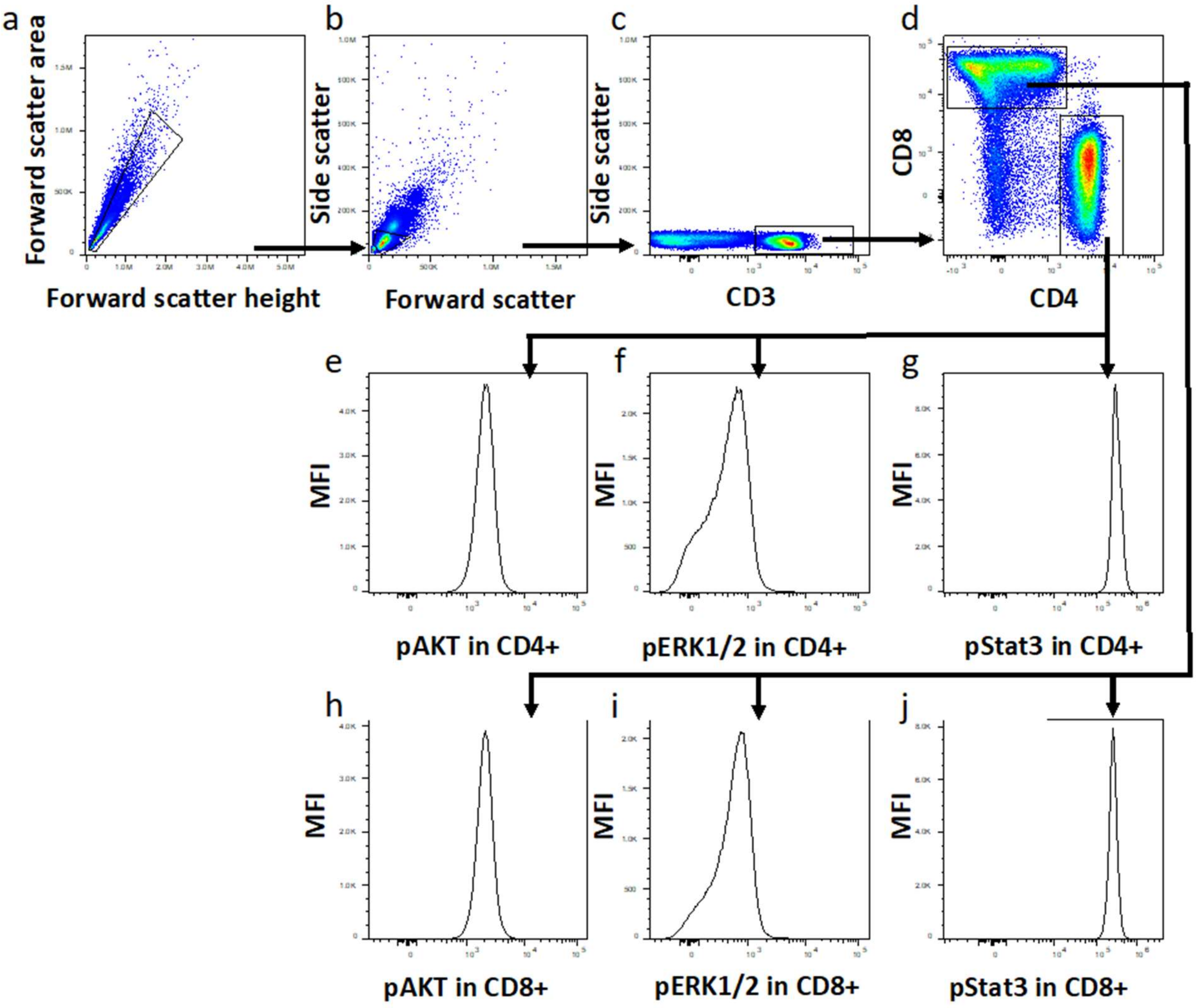

Figure 7. Gating of phosphospecific flow cytometry. A: Cells were gated on an FSC-H and FSC-A plot to eliminate doublets. B: Lymphocytes were gated on an FSC vs. SSC dot plot. C: CD3+ cells were chosen. D: CD4+ and CD8+ cells were separately gated. E-G: The MFI of pAKT, pERK1/2 and pStat3 in CD4+ was analysed. H-J: The MFI of pAKT, pERK1/2 and pStat3 in CD8+ was analysed. FSC: Forward scatter. FSC-H: Forward scatter height. FSC-A: Forward scatter area. SSC: Side scatter. MFI: Mean fluorescence intensity. pAKT: phosphorylated AKT. pERK1/2: phosphorylated ERK1/2. pStat3: phosphorylated Stat3.

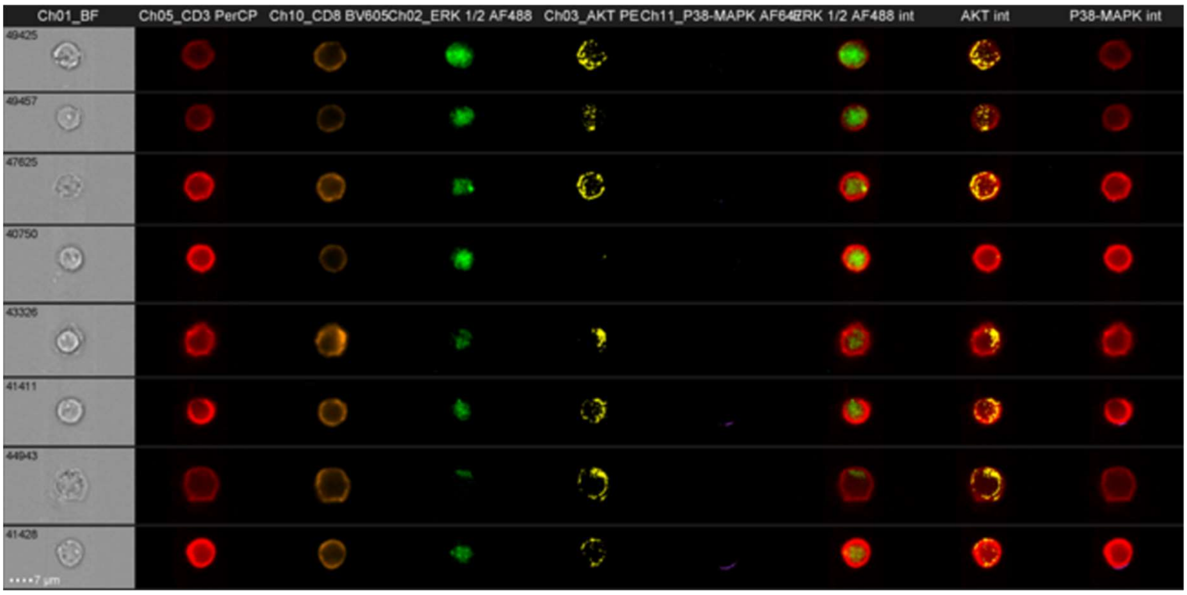

Figure 8. Verification of Intracellular staining using ImageStream®X (Amnis®). Shows where the fluorochromes brighten, thus where the corresponding antibody is placed. Prior to this table the cells were gated for CD3+ and CD8+ and as shown both CD3 and CD8 brighten up as expected on the surface of the cells. The figure shows that the fluorochromes for pERK1/2 and pAKT are positioned intracellularly as expected. Furthermore, it reveals very few cells containing p-p38 MAPK, thus it was excluded from our analysis. pERK1/2: phosphorylated ERK1/2. pAKT: phosphorylated AKT. p-p38 MAPK: phosphorylated p38 MAPK.

Table 1. Shows the flow cytometry cell panel with the different fluorochromes conjugated to which antibody and which clone was used. Each row represents one 5-mL BD Falcon™ tube analysed in the Novocyte flow cytometer (ACEA Biosciences, Inc.). All antibodies were obtained from BD Biosciences unless specified otherwise. All volumetric specifications are μL from the specific antibody, when stated <1μL it was diluted in bovine specific antigen (BSA) to be able to add the small amount of antibody. * PE instead of AF647 was used. ** FITC instead of AF488 was used. *** 0.5μL CD19-FITC clone HIB19, 0,5μL CD3-FITC clone UCHT1, 2μL CD14-FITC clone HCD14, 1,7μL CD56-FITC clone HCD56. # Obtained from Biolegend. ## A blue 5mL Polypropylene tube (Beckman Coulter) instead of 5-mL BD Falcon™ tube was used.

Table S1. Flow panel and clones used.

**Laser**							
Cells	PE	BV421	Alexa Fluor488	AF647	PerCP	BV786	BV605
Th1/17	7μL, CCR5, clone 3A9	2μL, CCR6, clone 11A9		10μL, CD45RO, clone UCHL1 *	1μL, CD3, clone SK7#	1,7μL, CD4, clone SK3	2,5μL CXCR3, clone IC6
Tregs		2μL, CD25, clone M-A251	1μL, CD4, clone RPA-T4**#	10μL, FoxP3, clone 259D#			
Tfh	20μL, ICOS, clone DX29	1,5μL, CXCR5, clone RF8B2	1μL, CD4, clone RPA-T4**#		1μL, CD3, clone SK7#		

Table S1. Flow panel and clones used.

**Laser**							
Monocytes[##]	20µL, AKT, clone M89-61	2µL, Stat3, clone 49/p-Stat3	20µL, ERK1/2, 20A	5µL, p38 MAPK, 36/p38		5µL, CD14, M5E2	5µL, CD16, clone 3G8
Th-Tc	20µL, AKT, clone M89-61	2µL, Stat3, clone 49/p-Stat3	20µL, ERK1/2, 20A	5µL, p38 MAPK, 36/p38	1µL, CD3, clone SK7[#]	3µL, CD4, clone SK3	5µL, CD8, clone SK1
Laser							
Cells	PE	BV421	FITC	APC	PerCp-Cy5.5	PE-Cy7	
DCs	0,5µL, ILT3, clone ZM4.1[#]	5µL, HLA-DR, clone L243[#]	CD19/3/14/56***[#]	0,17µL, CD86, clone IT2.2[#]	2,5µL, CD11c, clone 3.9[#]	1µL, CD123, clone 6H6[#]	
Monocytes[##]			2µL, CD14, clone HCD14[#]				

© 2019 by the authors. Submitted for possible open access publication under the terms and conditions of the Creative Commons Attribution (CC BY) license (http://creativecommons.org/licenses/by/4.0/).
